# Actuator Transcription Factors Resolve Combinatorial Binding Into Productive Gene Expression: An Evolutionary and Functional Framework for Gene Activation by Combinatorial TF Assemblies

**DOI:** 10.1002/bies.70198

**Published:** 2026-10-23

**Authors:** Nicoletta Bobola, Zoulfia Darieva

**Affiliations:** ^1^ Faculty of Biology, Medicine, and Health University of Manchester Manchester UK

## Abstract

Gene expression programs are central to life, as they establish and preserve cell identity during embryonic development and maintain tissue function in adulthood. When disrupted, they can give rise to a wide range of diseases. Precise gene regulation depends on the combinatorial binding of transcription factors (TFs) at enhancers, yet how these assemblies convert DNA binding into productive transcription remains poorly understood. Here, we propose that productive combinatorial binding can involve a distinct functional class of TFs, which we term transcriptional actuators. Using MEIS proteins as a paradigm, we define the core features that distinguish actuator TFs from other regulatory factors. Actuators are broadly expressed, sequence‐specific TFs that function predominantly as transcriptional activators and execute transcriptional programs specified by lineage‐restricted TFs. Finally, we outline experimental strategies to test actuator function, discuss evolutionary. implications, and explore how incorporating actuator TFs may enhance cellular reprogramming and regenerative strategies.

## Graphical abstract

Actuators convert combinatorial transcription factor (TF) binding into productive transcription. Broadly expressed actuators are recruited to enhancers by cooperating lineage‐restricted TFs and mediate transcriptional activation. Two enhancer scenarios: lineage‐specific selector TFs bound to an enhancer alone (light blue) do not activate transcription (OFF), whereas TF assembly with an actuator TF (red) enables transcription (ON).

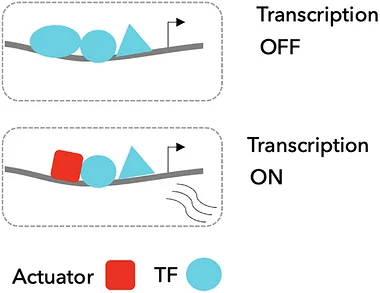

## 1 Introduction

Gene expression programs determine and maintain cell fate both in embryonic development and adult tissue homeostasis, and their dysregulation causes a broad range of diseases. Gene expression is largely controlled by TFs, which bind enhancers and recruit co‐activators to activate RNA Polymerase II at gene promoters [1].

TFs contain highly conserved DNA‐binding domains that recognize short (5–11 bp) DNA motifs [2]. These motifs are abundant and often shared among TFs of the same family, so on their own they provide little information for precise enhancer targeting [3]. Chromatin converts this low intrinsic specificity into precise gene regulation. Nucleosomes restrict DNA accessibility, and individual TFs rarely bind productively in isolation [4]. Instead, when multiple TFs bind adjacent motifs within an enhancer, they can cooperatively destabilize nucleosomes and stabilize each other's binding [5, 6]. A recent study suggests that binding cooperativity arises from TF activation domains recruiting cofactors that destabilize nucleosomes [7]. This chromatin‐imposed requirement for cooperative assembly acts as a selective filter: the TF combinations present in the appropriate cellular context can productively engage only a specific subset of enhancers (those with the correct arrangement of recognition motifs) and activate transcription. In this way, cooperativity sharpens TF selectivity and expands the regulatory capacity of a limited TF repertoire, enabling precise control during development and cellular reprogramming.

What remains unresolved is *how these TF‐assemblies function after binding*. While it is well established that multiple TFs facilitate each other's binding at the same enhancer, little is known about the subsequent steps: how do such constellations of TFs modulate RNA polymerase II activity? Are they functionally redundant in recruiting co‐activators, or do they contribute distinct and non‐interchangeable roles in transcriptional activation?

We propose that functional instances of combinatorial binding, namely those that lead to transcriptional activation, may involve TFs with qualitatively distinct roles. One possible solution may be the use of transcriptional “actuators”. Actuators are broadly expressed across tissues and function to fully execute transcriptional programs that are specified by context‐specific TFs. We propose that actuators represent one regulatory architecture through which combinatorial TF binding is converted into productive transcription.

We will present supporting evidence for this hypothesis, drawing largely on studies of three amino acid loop extension (TALE) TFs, and describe a *guided activation* model of transcription, its evolutionary significance, and its potential applications.

## 2 MEIS TFs as Transcriptional “Actuators”

TALE homeodomain (HD) superclass comprises an ancient, evolutionarily conserved group of TFs distinguished by an atypical homeodomain with a three‐amino‐acid insertion between helix 1 and helix 2 [8, 9, 10]. TALE factors are highly conserved across metazoans. In humans, they include the MEIS (MEIS1–3), PBX (PBX1–4), and PKNOX (PKNOX1/2, also known as PREP1/2) families, while in Drosophila, their functional counterparts are homothorax (hth, MEIS homolog) and extradenticle (exd, PBX homolog). MEIS proteins (Myeloid Ecotropic Viral Integration Site) were initially identified as required for HOX‐mediated transformations in a mouse model of leukemia [11, 12]. Beyond their classical role as HOX partners, MEIS TFs are essential for development of multiple organs, including the limb, face, eye, and heart, where they regulate gene expression programs that guide cellular differentiation and morphogenesis [13]. In addition, MEIS proteins can act as oncogenes, with dysregulated expression contributing to tumorigenesis in diverse tissues [13].

### 2.1 MEIS Binding Is Associated With Tissue‐Specific TFs

Genes encoding for MEIS TFs are broadly expressed (Figure 1A) [14], yet numerous observations link protein activity to tissue‐ and lineage‐specific transcriptional programs. Across the branchial arches (BAs), a series of metameric, transient tissues of vertebrate embryos, MEIS TFs bind to largely overlapping locations [15]. However, high MEIS binding levels (“top peaks”) occur at locations that are specific for each BA and are associated with increased enhancer activity and higher gene expression in the corresponding tissue (Fig. 1BC) [15]. These observations suggest that MEIS TFs contribute to each BA‐specific transcriptional program. Indeed, top MEIS peaks in each BA are enriched for motifs recognized by distinct, tissue‐specific TFs (Figure 1D) [15, 16].

**FIGURE 1:**
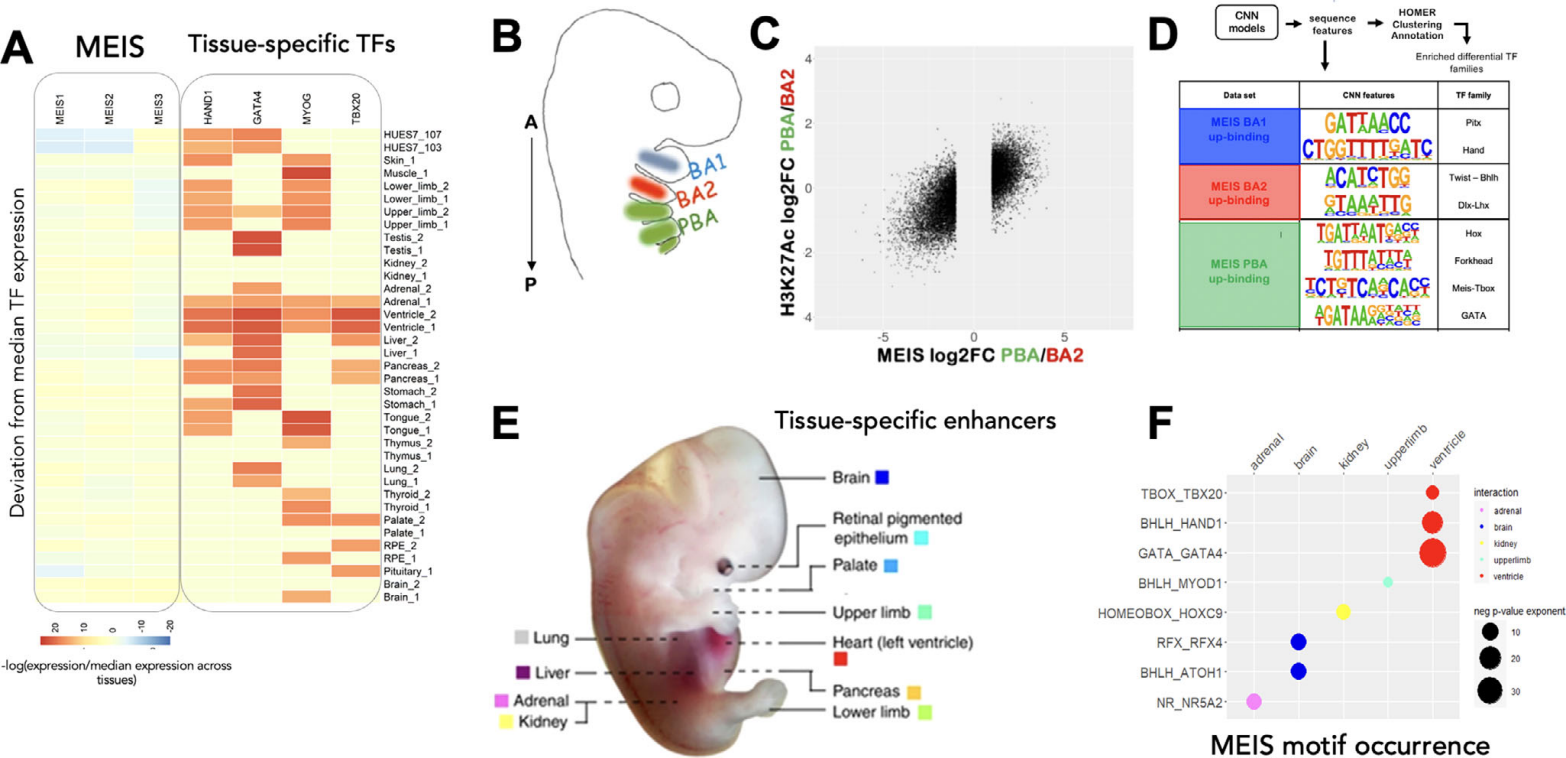
MEIS binding is associated with tissue‐specific TFs. (A). Left panel: expression of *MEIS1*‐2‐3 in human embryonic stem cells (HUES7) and tissues. Right panel shows examples of tissue‐restricted TFs. All tissues are duplicated except for pituitary, muscle, and skin. Human embryonic RNA‐seq read counts for each gene were divided by the gene's median read counts. Negative logarithms of the median normalized counts were used to plot the heatmap (adapted from [14], licensed under Creative Commons Attribution 4.0 International (CC BY 4.0): https://creativecommons.org/licenses/by/4.0/). Median normalized counts are reported in Table 1. (B). Schematic representation of a mouse embryo, showing colour‐coded branchial arches along the AP (antero‐posterior) axis (blue = BA1; red = BA2; green = posterior BA, including BA3‐6). (C). Correlation plot of differential MEIS binding and differential acetylation (enhancer activity) at intergenic regions in PBA versus BA2. Each point corresponds to a region with differential MEIS binding log2 fold change >1 (FC>2); the corresponding differential H3K27ac value is plotted. Changes in MEIS binding levels are positively correlated with increased enhancer activity in the same tissue (correlation = 0.73). (adapted from [15], licensed under CC BY 4.0). (D). MEIS binding is classified in three classes (i.e. peaks with higher binding in BA1, BA2, PBA). Convolutional neural networks (CNNs) models of MEIS differential peaks identify tissue‐specific enrichment of sequence motifs in each class of MEIS peaks (adapted from [15], licensed under CC BY 4.0). (E). Human embryo showing the tissue/organs used to identify tissue‐specific active (H3K27Ac‐positive) regions; samples collected ranged from Carnegie Stage 14 to 21 (adapted from [14, 17], licensed under CC BY 4.0). (F). TALE motif co‐occurs with motifs enriched in tissue‐specific enhancers active in human embryonic development. The tissue‐restricted TFs listed on the left are predicted to bind signature motifs enriched in human enhancers specifically active in the tissue shown. TALE motif was identified as enriched nearby (100 nt upstream or downstream) the tissue‐restricted TF motif. A cutoff of *p* < 1 × 10^−5^, calculated by HOMER using a cumulative binomial distribution, and enrichment in >5% of foreground regions was used. The size of the bubbles matches the negative log *p* value: larger bubbles represented smaller *p* values (adapted from [14, 17], licensed under CC BY 4.0).

**TABLE 1:** Median RNA counts across tissues, with counts down‐sampled to the 75th percentile within each tissue, for genes encoding the TFs shown in Figure 1A.

	Ensembl. ID	HGNC. symbol	Median
MEIS3	ENSG00000105419.17	MEIS3	500
HAND1	ENSG00000113196.2	HAND1	4
MYOG	ENSG00000122180.4	MYOG	8
MEIS2	ENSG00000134138.19	MEIS2	1293
GATA4	ENSG00000136574.17	GATA4	8
MEIS1	ENSG00000143995.19	MEIS1	1285
TBX20	ENSG00000164532.10	TBX20	3

A screen mapping combinatorial TF binding in human developmental enhancers [17], also highlighted the potential contribution of MEIS TFs to tissue‐specific gene expression programs. In enhancers clustered by tissue‐specific activity (Figure 1E), TALE TF recognition motifs were found highly enriched near tissue‐specific sequence signatures. TALE motifs often occurred adjacent to motifs recognized by tissue‐restricted TFs in enhancers active in the heart (ventricle), brain, upper limb, adrenal gland, and kidney (Figure 1F). In contrast to the broadly expressed MEIS TFs, their potential co‐binding partners are generally tissue‐restricted.

In sum, high confidence MEIS binding (high binding levels) displays two main features: enhancer activity and tissue‐specificity. These findings highlight a distinctive behavior for MEIS TFs, and suggest a ‘general’ role in gene activation.

### 2.2 Tissue‐Specific TFs Shape MEIS Occupancy

The consistent association of MEIS binding with motifs for tissue‐specific TFs suggests functional collaboration. This is supported by experimental evidence. In branchial arch 2 (BA2), HOXA2 specifically enhances MEIS occupancy at regions containing HOX motifs. These high HOXA2–MEIS peaks are strongly linked to HOXA2 activated genes, suggesting that HOXA2 switches its transcriptional program by increasing or stabilizing binding of MEIS TFs [18]. During cardiomyocyte differentiation, HOXB1 promotes MEIS binding at its target enhancers. MEIS1 also co‐binds with GATA TFs (GATA4‐6) at cardiac enhancers. Increasing HOXB1 levels redistributes MEIS1 binding from GATA‐ to HOX‐dependent enhancers, accompanied by corresponding transcriptional changes in GATA‐ and HOX‐regulated genes [14]. Similar cooperation of MEIS proteins and tissue‐specific TFs is seen in multiple systems. In projection neurons, MEIS2 is guided to enhancers associated with projection‐neuron‐specific genes by the homeodomain TF DLX5 [19]. In the retina, MEIS TFs collaborate with the homeodomain TF LHX2 to activate the expression of retinal progenitor cell‐specific genes [20]. MEIS TFs cooperate with HOX and TBX5 to establish early limb development [21], and with HOXB13 in postnatal cardiomyocyte maturation and proliferation [22]. In sum, these findings indicate that tissue‐specific TFs shape MEIS occupancy, revealing a general principle: MEIS binding is guided by tissue‐specific TFs.

### 2.3 MEIS TFs Mainly Function as Transcriptional Activators

The consistent pairing of MEIS with context‐specific TFs suggests that cooperation may go beyond simple binding facilitation, hinting at a broader regulatory role. Which regulatory function MEIS may provide that is selectively exploited by lineage‐defining factor?

Several lines of evidence indicate that MEIS TFs are predominantly associated with activation of transcription. Across diverse embryonic tissues, differential binding of MEIS positively correlates with enhancer activity, indicating a role for MEIS in the activation of gene expression [15, 17]. In cardiac progenitors, MEIS TFs predominantly function as transcriptional activators, promoting chromatin accessibility and acetylation at critical regions for cardiac differentiation, and the expression of their associated genes [14]. In hematopoietic cells, HOXA9 and MEIS1 co‐occupy distal enhancer sites marked by H3K4me1 and p300/CBP, consistent with an activation role at enhancers [23]. In mouse embryonic stem cells, where HOXB1 largely cooperate with the repressor REST, the fraction of HOXB1 and PBX/MEIS co‐occupied sites correlate instead with active chromatin marks and gene activation [24]. This genomic evidence supports a model where MEIS predominantly marks active enhancers.

Biochemical and functional analyses have narrowed down transactivation function to the C‐terminal domain in MEIS1. *MEIS1* acts as an oncogene in acute myeloid leukemia (AML), and its overexpression accelerates HOXA9‐driven AML [25, 26]. MEIS1 transforming activity requires the protein's C‐terminal domain (CTD). Adding the VP16 transactivation domain (a potent transcriptional activator) to MEIS mutants lacking the CTD can restore MEIS transforming function, linking the oncogenic properties of MEIS1 to the ability to activate gene expression [27, 28, 29, 30]. Additional support for MEIS role in transcriptional activation comes from genetic studies of Homothorax (Hth), the Drosophila homolog of vertebrate MEIS. In Drosophila, converting Hth into a repressor (by fusing Hth to the Engrailed repression domain) mimics Hth loss‐of‐function, suggesting that Hth normal function is to activate downstream target genes [31].

Despite the clear link with activation of gene expression, MEIS proteins do not behave as classical trans‐activators in reporter assays: overexpression of MEIS2 together with target enhancers or promoters generally results in minimal or no reporter activation [19]. This observation can be partly explained by the finding that, in the MEIS2 protein, the Hth domain inhibits the activity of the activation domain [32]. In addition, MEIS TFs bind predominantly to distal regulatory regions rather than promoters [15, 18], suggesting that MEIS activates transcription over long distances by recruiting chromatin‐associated proteins. Thus, MEIS function may become apparent only in a chromatinized context.

### 2.4 MEIS Function Converges on Enhancer Activation

Based on the widespread association of MEIS1 binding with transcriptional activation across diverse tissues, a likely hypothesis is that MEIS TFs execute a shared, tissue‐independent function, essential for gene activation. Consistent with this idea, in zebrafish embryos Meis (together with Hox) displaces HDACs and promotes histone acetylation at Hox target promoters, providing a direct mechanistic link to transcriptional activation [33].

Further support for a general activating role of MEIS comes from human genetics. Mutations in *MEIS2* cause a rare developmental disorder (MEIS2 syndrome) characterized by craniofacial abnormalities, developmental delay, intellectual disability, and cardiac and skeletal defects [34, 35]. Notably, these features closely resemble those of Kabuki syndrome [36], which is most commonly caused by mutations in *KMT2D* [37]. *KMT2D* encodes a ubiquitously expressed histone‐lysine methyltransferase that deposits H3K4me1 at distal enhancers, thereby promoting enhancer activation [38]. Because KMT2D lacks a DNA‐binding domain, it depends on sequence‐specific TFs for genomic targeting. Several observations support MEIS TFs as plausible recruiters of KMT2D to context‐specific enhancers. Like *MEIS1–2*, *KMT2D* is required for cardiac development [39], and dysregulation of both MEIS1 and MLL‐family KMT2 proteins contributes to the pathogenesis of AML. Two independent observations support a mechanistic link between MEIS and KMT2D. First, MEIS1 physically associates with KMT2D (also known as MLL4) in AML cell lines, where KMT2D ranks among the top MEIS1 interactors [40]. Second, during differentiation of human embryonic stem cells into cardiac lineages, MEIS1 and KMT2D show partially overlapping genomic occupancy, and KMT2D binding is selectively reduced at MEIS‐bound regions (but not at other genomic sites) in the absence of MEIS1‐2 [14]. Collectively, these findings support a model in which MEIS TFs activate enhancers through recruitment of the methyltransferase KMT2D, promoting enhancer commissioning during lineage specification and providing a mechanistic basis for MEIS function as a transcriptional activator.

Nevertheless, additional evidence is required to establish conclusively that MEIS‐dependent transcriptional activation is mediated primarily through KMT2D. It also remains unclear whether MEIS activity relies exclusively on KMT2D recruitment or whether MEIS factors operate via multiple activating mechanisms. Resolving these questions will be important not only for understanding MEIS function, but also for clarifying the shared molecular basis of MEIS2 and Kabuki syndromes.

## 3 Actuators and the Guided Activation Model

Based on the observations made on MEIS TFs, we define actuator TFs as a distinct functional class, with four defining characteristics. First, actuators are **broadly expressed**, yet unlike ubiquitous TFs (e.g. SP1 [41], NFY) [42], they do not impose a uniform gene expression output across cell types. Second, actuators **contribute to distinct, tissue‐specific transcriptional programs** through selective collaboration with lineage‐restricted transcription factors. Third, they **function predominantly as transcriptional activators**. Finally, actuators **are sequence‐specific DNA‐binding proteins**, distinguishing them from co‐activators (e.g. p300/CBP or mediator) [43], which typically lack intrinsic DNA‐binding capacity and must be recruited by TFs.

Actuator TFs share some properties with pioneer TFs, including broad expression, cooperation with lineage‐specific factors, and association with enhancer activation. However, while pioneer factors are defined by their ability to engage nucleosomal DNA and establish chromatin accessibility at previously inaccessible regulatory regions, actuators are defined by their ability to convert enhancer occupancy into productive transcriptional activation.

Actuators are directed to specific enhancers through combinatorial binding with lineage‐specific TFs [14]. In this model, actuators and lineage‐specific TFs recognize nearby motifs in enhancers. Co‐binding stabilizes TF‐DNA interactions and increases actuator residence time at target enhancers, thereby promoting local co‐activator recruitment and activation of gene expression (Figure 2A). We refer to this framework as *guided activation*.

**FIGURE 2:**
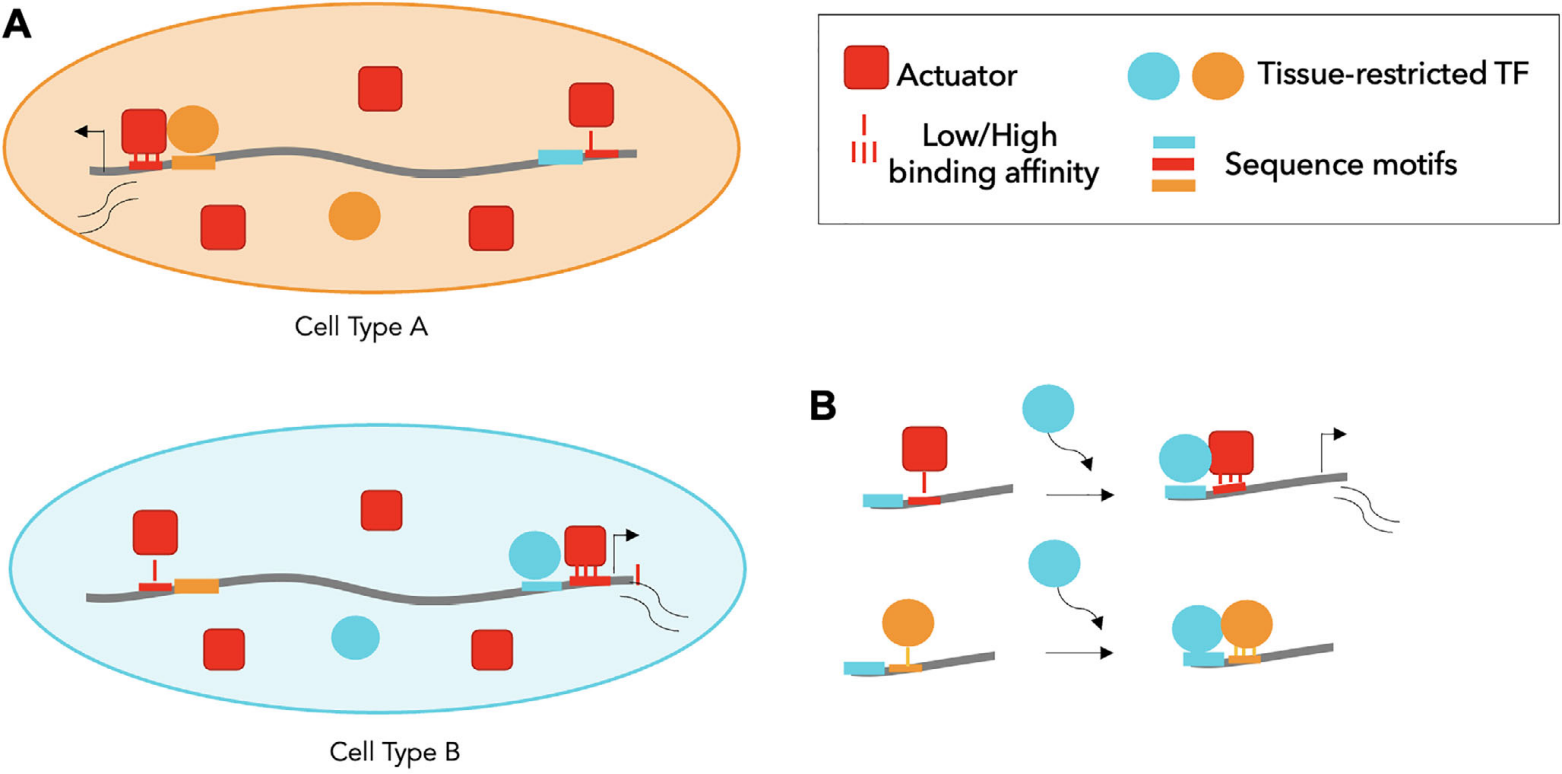
Actuator TFs and the guided activation model. (A). Lineage‐specific TFs guide actuators to selected enhancers, a mechanism termed *guided activation*. Selected enhancers contain motifs for actuator and cell type‐specific TF (marked by red+orange and red+blue motifs). Binding of cell type‐specific TFs (orange in cell type A and blue in cell type B) locally amplifies MEIS occupancy at selected enhancers, resulting in cell type‐specific gene expression in cell types A and B. Cell‐type specific enhancers show low MEIS binding in the absence of the cell type‐specific TF (e.g., MEIS binds weakly to a cell type B enhancer in cell type A). MEIS TFs function as a reusable enhancer actuator across tissues. (B). Actuators (red) bind DNA weakly and transiently on their own. Co‐binding with lineage‐specific TFs (blue) prolongs their residence time at target enhancers, promoting local co‐activator recruitment, and activates gene expression. In contrast, when the lineage‐specific TFs (blue) stabilize the binding of a non‐actuator TF (orange), this does not lead to gene activation.

DNA binding alone does not necessarily lead to transcriptional activation, and TFs differ substantially in their capacity to activate transcription. TFs are traditionally viewed as modular proteins composed of a DNA‐binding domain and an effector domain. In contrast to the well‐structured DNA‐binding domain, effector domains are intrinsically disordered and lack conserved sequence features that enable straightforward identification [44]. Recently, scalable functional assays have been implemented to identify effector domains in yeast [45, 46, 47], Drosophila [48], and human cells [49, 50]. Across systems, these approaches consistently show that robust activation capacity is rare among TFs. Supporting this conclusion, systematic measurements of TF activity reveal large differences in intrinsic activation strength among DNA‐bound TFs [7]. Collectively, these observations are consistent with the guided activation model, in which transcriptional activation is not an intrinsic property of most DNA‐binding TFs but is instead concentrated in a distinct functional subset with strong activation potential.

A prediction of the guided activation model, and the division of labor between broadly expressed activators and lineage‐restricted factors, is that DNA‐bound TF assemblies are more likely to be functional if they include at least one actuator or a TF with strong intrinsic activation potential (Fig. 2AB). This requirement provides a rationale for why only a small subset of the many TF‐DNA interactions detected by ChIP‐seq results in changes in gene expression [51]. Given the limited sequence specificity of most TFs, gene expression would otherwise be widespread and nonspecific if activation capacity were a general property shared by most TFs.

Actuators may function as shared, and potentially limiting, regulatory hubs if multiple lineage‐specific TFs converge on a common activating factor. A key prediction is that changes in the abundance or activity of either the actuator or its recruiting TF partners can redistribute actuator occupancy and consequently alter the balance between competing transcriptional programs. Whether actuator availability is limiting in all contexts remains an open question; however, experimental evidence from cardiac differentiation supports this prediction. During cardiac differentiation, MEIS occupies both HOX‐ and GATA‐dependent enhancers, and the relative contribution of these regulatory inputs changes over time. Increasing HOXB1 levels experimentally increases MEIS occupancy at HOX‐dependent enhancers while reducing MEIS occupancy at GATA‐dependent regions, accompanied by altered expression of HOX‐ and GATA‐regulated genes and delayed cardiac differentiation [14]. These observations are consistent with a model in which lineage‐specific TFs compete for access to a shared actuator pool, allowing changes in one regulatory input to propagate through the broader transcriptional network.

The evolution of multicellularity required mechanisms to achieve context‐specific gene regulation. TALE TFs are evolutionary ancient and were present in unicellular eukaryotes prior to the diversification of metazoans [52, 53]. These ancestral TFs likely functioned as general transcriptional activators, coupling DNA binding to gene activation in relatively simple regulatory contexts. With the emergence of multicellularity and increasing cellular specialization, regulatory specificity expanded through the progressive evolution of lineage‐restricted DNA‐binding factors, such as HOX proteins, that could exploit these pre‐existing activators to achieve cell type‐specific gene regulation. In this view, guided activation reflects an evolutionarily strategy in which ancient actuator TFs provide the activation potential, while lineage‐specific TFs confer specificity by directing actuators to selected enhancers. Rather than evolving new activation domains, multicellular organisms repurposed a small set of pre‐existing activators. This framework further suggests that additional actuator TFs may exist among known DNA‐binding proteins; their identification will be critical for understanding how enhancer binding translates into transcriptional output across diverse biological contexts.

### 3.1 Testing the Actuator Hypothesis

Testing the actuator hypothesis requires addressing two central questions: first, how actuator TFs causally couple DNA binding to transcriptional activation; and second, whether this function is both necessary and sufficient for productive gene expression. Dissecting causality is complicated by the substantial gaps that remain in our understanding of transcriptional regulation. Although thousands of TFs and chromatin‐associated proteins have been identified, the causal sequence of events linking distal TF binding at enhancers to RNA polymerase II recruitment and activation at promoters remains poorly understood. Several models have been proposed, including enhancer–promoter looping, condensate formation, and phase separation [54], yet no single framework has emerged as universally applicable across loci or cell types. The prevailing paradigm is that TFs recruit general coactivators or corepressors that, in turn, recruit or stabilize RNA polymerase II at promoters. Assuming that actuators—like other TFs—do not possess intrinsic activation capacity, identifying proteins that co‐occupy chromatin with MEIS represents a key starting point for elucidating MEIS function. This could be achieved using chromatin‐based immunoprecipitation approaches coupled to mass spectrometry, such as RIME [55], or proximity‐based labelling, such as BioID and its derivatives [56].

Subsequent manipulation of the MEIS–coactivator interface would allow direct testing of whether transcriptional activation can be predictably gained or lost as a consequence of these perturbations. Key questions include whether actuators are required for transcriptional activation at their bound sites, whether their recruitment is sufficient to convert non‐productive binding events into productive ones, and whether actuator occupancy quantitatively scales transcriptional output in a context‐dependent yet predictable manner. Finally, an important open question is whether additional actuators exist among known TFs, and if so, which factors fulfil this role.

### 3.2 Are There Additional Transcriptional Actuators?

The human TF repertoire includes about 1,600 known or putative TFs [2]. Identifying actuator TFs requires defining their distinguishing mechanistic features. Because these features are not fully elucidated, the four characteristics outlined above provide a useful working framework.

Most TFs are DNA‐binding proteins and many, similar to MEIS, are broadly expressed yet drive highly tissue‐specific programs. These include members of small families, such as TEAD [57], RUNX [58], and GATA [59], as well as larger families, such as SP/KLF [41], and bZIP. Despite these shared properties, their functional outputs differ substantially.

A defining feature of MEIS is its predominant role as a transcriptional activator, with no evidence for widespread repressive activity. In contrast, many TFs, including those listed above, can function as either activators or repressors depending on cellular context, often through the recruitment of corepressor complexes. Estimates of how frequently such bifunctionality occurs vary across studies: one large‐scale analysis reported dual functionality to be widespread [60], whereas a distinct screening framework identified bifunctional activity in only ∼12% of tested proteins [61]. These discrepancies likely reflect differences in experimental context, highlighting the context dependence of TF regulatory activity.

Why the flexibility to act as activators or repressors appears to be relatively common in TFs, sometimes even simultaneously [62], whereas MEIS is strongly biased toward activation remains unclear. Defining the mechanistic properties that underlie MEIS function will be essential for understanding this distinction. There are partial parallels between MEIS and other TFs. For example, SP1 functions predominantly as a transcriptional activator and regulates tissue‐specific programs, although instances of repression have been reported. Unlike MEIS, however, SP1 also controls broadly shared transcriptional programs, suggesting that it can operate independently of lineage‐specific or guiding TFs. Moreover, SP1 primarily acts through GC‐rich promoters while retaining some capacity for distal binding [41].

PBX proteins, which like MEIS belong to the TALE family, share several core mechanistic features with MEIS. Both bind DNA weakly on their own and depend on both tissue‐specific partners (e.g., HAND2, MYOD) [63, 64] and more ubiquitous factors (e.g., NFY) [65] to achieve stable chromatin occupancy. In this respect, PBX may function similarly to MEIS as an actuator TF, integrating contextual cues to drive specific transcriptional outputs. However, PBX proteins extend this logic by acting earlier in development: PBX expression is detected at the earliest developmental stages, preceding zygotic genome activation [66], and PBX has been proposed to function as a pioneer factor capable of engaging chromatin prior to widespread transcriptional competence [64, 67]. In contrast, MEIS expression initiates later, coincident with zygotic genome activation, suggesting that MEIS operates primarily within chromatin landscapes that have already been at least partially established.

The identification of additional actuator TFs will be essential both to establish the generality of this model and to define how enhancer binding is translated into transcriptional output across diverse biological contexts.

## 4 Conclusions and Perspectives

The guided activation framework represents one model of enhancer architecture that may explain how combinatorial TF binding is converted into tissue‐ and cell type‐specific gene activation. It is well established that multiple TFs facilitate one another's binding at the same enhancer, yet how these TF‐assemblies function after binding remains poorly understood. Guided activation proposes that TFs differ in their ability to activate transcription. Based on our observations of TALE TFs, we propose a division of labor between lineage‐enriched TFs, which select cell type‐specific enhancers, and broadly expressed actuator TFs, which activate enhancer function and drive gene expression. In this model, an actuator TF is recruited to selected regulatory regions through cooperation with context‐specific TFs, providing a mechanism through which enhancer selection and transcriptional activation can be coupled.

The framework generates testable predictions. Actuator occupancy should depend on the abundance and activity of its recruiting TF partners, while changes in actuator availability may redistribute transcriptional activity across competing regulatory programs. Actuators may therefore represent central integration points in gene regulatory networks, translating selected combinations of TFs into productive transcriptional outputs.

More broadly, the division of labor between actuators and context‐specific TFs provides a framework for decoding combinatorial “binding codes”. The key question is not simply which TFs bind an enhancer, but which combinations of TFs include an actuator capable of driving transcription. Understanding these relationships may reveal how cell type‐specific TF combinations are translated into enhancer activity, and ultimately enable us to predict which combinations of motifs are functional in a particular cellular context.

Because MEIS—the prototype for these actuator factors—is evolutionarily ancient, it is tempting to speculate that guided activation emerged alongside multicellularity and the evolution of lineage‐specific transcription factors. If so, this mechanism has been remarkably well conserved. We do not propose that guided activation is the only possible enhancer architecture. Rather, it may represent one solution to the challenge of achieving cell type‐specific gene expression. An alternative may rely on the cooperative assembly of multiple TFs with relatively weak activation potential to achieve robust enhancer activation without dedicated actuator proteins.

Framing transcriptional regulation around actuators also offers new perspectives on how cell states are established and may inform reprogramming strategies. Predominantly transcriptional activators with intrinsic DNA‐binding capacity, actuators could implement tissue‐specific gene programs when recruited by lineage‐specific TFs. Lineage‐specific TFs alone often yield limited efficiency in generating desired cell types [68]. Integrating broadly expressed actuators could therefore enhance these protocols by amplifying lineage‐specific programs and stabilizing cell identity. Understanding the sequence features that encode actuator function may further enable the engineering of natural or synthetic TFs with customized reprogramming capabilities.

Finally, TFs operate within highly interconnected networks that define cell identity, and perturbations in these networks underlie diverse diseases, including cancer, developmental disorders, and cardiovascular disease. Broad activators, in particular, may exert pervasive transcriptional effects and may include oncogenes. Systematically identifying actuator TFs across medically relevant cell types could therefore advance our understanding of disease mechanisms and inform strategies for diagnosis, personalized therapy, and regenerative medicine.

## Author Contributions

NB and ZD jointly developed the concept and scope of the article. NB drafted the manuscript and created the figures. ZD edited and revised the manuscript. Both authors read and approved the final version of the manuscript prior to submission.

## Conflicts of Interest

The authors declare no conflicts of interest.

## Data Availability

Data sharing not applicable to this article as no datasets were generated or analyzed during the current study.

## References

[1] F. Spitz and E. E. Furlong , “Transcription Factors: From Enhancer Binding to Developmental Control,” Nature Reviews Genetics 13 (2012): 613–626, 10.1038/nrg3207. 22868264

[2] S. A. Lambert , A. Jolma , L. F. Campitelli , et al., “The Human Transcription Factors,” Cell 172 (2018): 650–665, 10.1016/j.cell.2018.01.029. PMC12908702 29425488

[3] S. Kim and J. Wysocka , “Deciphering the Multi‐scale, Quantitative Cis‐regulatory Code,” Molecular Cell 83 (2023): 373–392, 10.1016/j.molcel.2022.12.032. PMC9898153 36693380

[4] F. Reiter , S. Wienerroither , and A. Stark , “Combinatorial Function of Transcription Factors and Cofactors,” Current Opinion in Genetics & Development 43 (2017): 73–81, 10.1016/j.gde.2016.12.007. 28110180

[5] L. A. Mirny , “Nucleosome‐mediated Cooperativity Between Transcription Factors,” Proceedings of the National Academy of Sciences 107 (2010): 22534–22539, 10.1073/pnas.0913805107. PMC3012490 21149679

[6] G. Moyle‐Heyrman , H. S. Tims , and J. Widom , “Structural Constraints in Collaborative Competition of Transcription Factors Against the Nucleosome,” Journal of Molecular Biology 412 (2011): 634–646, 10.1016/j.jmb.2011.07.032. PMC3534743 21821044

[7] B. R. Doughty , M. M. Hinks , J. M. Schaepe , et al., “Single‐molecule States Link Transcription Factor Binding to Gene Expression,” Nature 636 (2024): 745–754, 10.1038/s41586-024-08219-w. PMC12326879 39567683

[8] N. Bobola and C. G. Sagerström , “TALE Transcription Factors: Cofactors no More,” Seminars in Cell & Developmental Biology 152–153 (2024): 76–84, 10.1016/j.semcdb.2022.11.015. 36509674

[9] T. R. Bürglin and M. Affolter , “Homeodomain Proteins: An Update,” Chromosoma 125 (2016): 497–521, 10.1007/s00412-015-0543-8. PMC4901127 26464018

[10] L. Selleri , V. Zappavigna , and E. Ferretti , “‘Building a Perfect Body’: Control of Vertebrate Organogenesis by PBX‐Dependent Regulatory Networks,” Genes & Development 33 (2019): 258–275, 10.1101/gad.318774.118. PMC6411007 30824532

[11] J. J. Moskow , F. Bullrich , K. Huebner , I. O. Daar , and A. M. Buchberg , “Meis1 , a PBX1 ‐Related Homeobox Gene Involved in Myeloid Leukemia in BXH‐2 Mice,” Molecular and Cellular Biology 15 (1995): 5434–5443, 10.1128/MCB.15.10.5434. PMC230793 7565694

[12] T. Nakamura , N. A. Jenkins , and N. G. Copeland , “Identification of a New family of Pbx‐related Homeobox Genes,” Oncogene 13 (1996): 2235–2242. 8950991

[13] D. Schulte and D. Geerts , “MEIS Transcription Factors in Development and Disease,” Development 146 (2019): dev174706, 10.1242/dev.174706. 31416930

[14] Z. Darieva , P. Zarrineh , N. Phillips , et al., “Ubiquitous MEIS Transcription Factors Actuate Lineage‐specific Transcription to Establish Cell Fate,” EMBO Journal 44 (2025): 2232–2262, 10.1038/s44318-025-00385-5. PMC12000411 40021842

[15] L. Bridoux , P. Zarrineh , J. Mallen , et al., “HOX Paralogs Selectively Convert Binding of Ubiquitous Transcription Factors Into Tissue‐specific Patterns of Enhancer Activation,” PLOS Genetics 16 (2020): 1009162, 10.1371/journal.pgen.1009162. PMC7769617 33315856

[16] M. Phuycharoen , P. Zarrineh , L. Bridoux , et al., “Uncovering Tissue‐specific Binding Features From Differential Deep Learning,” Nucleic Acids Research 48 (2020): 27, 10.1093/nar/gkaa009. PMC7049686 31974574

[17] A. Garcia‐Mora , J. Mallen , P. Zarrineh , N. Hanley , D. Gerrard , and N. Bobola , “Systematic Analysis of Transcription Factor Combinatorial Binding Uncovers TEAD1 as an Antagonist of Tissue‐Specific Transcription Factors in Human Organogenesis,” preprint *eLife* June 10, 2025.

[18] S. Amin , I. J. Donaldson , D. A. Zannino , et al., “Hoxa2 Selectively Enhances Meis Binding to Change a Branchial Arch Ground State,” Developmental Cell 32 (2015): 265–277, 10.1016/j.devcel.2014.12.024. PMC4333904 25640223

[19] E. Dvoretskova , M. C. Ho , V. Kittke , et al., “Spatial Enhancer Activation Influences Inhibitory Neuron Identity During Mouse Embryonic Development,” Nature Neuroscience 27 (2024): 862–872, 10.1038/s41593-024-01611-9. PMC11088997 38528203

[20] N. Dupacova , B. Antosova , J. Paces , and Z. Kozmik , “Meis Homeobox Genes Control Progenitor Competence in the Retina,” Proceedings of the National Academy of Sciences 118 (2021): 2013136118, 10.1073/pnas.2013136118. PMC7999952 33723039

[21] I. Delgado , G. Giovinazzo , S. Temiño , et al., “Control of Mouse Limb Initiation and Antero‐posterior Patterning by Meis Transcription Factors,” Nature Communications 12 (2021): 3086, 10.1038/s41467-021-23373-9. PMC8149412 34035267

[22] N. U. N. Nguyen , D. C. Canseco , F. Xiao , et al., “A Calcineurin–Hoxb13 Axis Regulates Growth Mode of Mammalian Cardiomyocytes,” Nature 582 (2020): 271–276, 10.1038/s41586-020-2228-6. PMC7670845 32499640

[23] J. Huang , M. Sabater‐Lleal , F. W. Asselbergs , et al., “Genome‐wide Association Study for Circulating Levels of PAI‐1 Provides Novel Insights Into Its Regulation,” Blood 120 (2012): 4873–4881, 10.1182/blood-2012-06-436188. PMC3520624 22990020

[24] N. P. Singh , B. De Kumar , A. Paulson , et al., “Genome‐Wide Binding Analyses of HOXB1 Revealed a Novel DNA Binding Motif Associated With Gene Repression,” Journal of Developmental Biology 9 (2021): 6, 10.3390/jdb9010006. PMC7931043 33546292

[25] E. Kroon , J. Krosl , U. Thorsteinsdottir , S. Baban , A. M. Buchberg , and G. Sauvageau , “Hoxa9 Transforms Primary Bone Marrow Cells Through Specific Collaboration With Meis1a But Not Pbx1b,” The EMBO Journal 17 (1998): 3714–3725, 10.1093/emboj/17.13.3714. PMC1170707 9649441

[26] U. Thorsteinsdottir , E. Kroon , L. Jerome , F. Blasi , and G. Sauvageau , “Defining Roles for HOX and MEIS1 Genes in Induction of Acute Myeloid Leukemia,” Molecular and Cellular Biology 21 (2001): 224–234, 10.1128/MCB.21.1.224-234.2001. PMC88796 11113197

[27] G. G. Wang , M. P. Pasillas , and M. P. Kamps , “Meis1 programs Transcription of FLT3 and Cancer Stem Cell Character, Using a Mechanism That Requires Interaction With Pbx and a Novel Function of the Meis1 C‐terminus,” Blood 106 (2005): 254–264, 10.1182/blood-2004-12-4664. PMC1895124 15755900

[28] A. Mamo , “Molecular Dissection of Meis1 Reveals 2 Domains Required for Leukemia Induction and a Key Role for Hoxa Gene Activation,” Blood 108 (2006): 622–629, 10.1182/blood-2005-06-2244. 16469876

[29] R. Bisaillon , B. T. Wilhelm , J. Krosl , and G. Sauvageau , “C‐terminal Domain of MEIS1 Converts PKNOX1 (PREP1) Into a HOXA9‐collaborating Oncoprotein,” Blood 118 (2011): 4682–4689, 10.1182/blood-2011-05-354076. 21900201

[30] G. G. Wang , M. P. Pasillas , and M. P. Kamps , “Persistent Transactivation by meis1 Replaces Hox Function in Myeloid Leukemogenesis Models: Evidence for Co‐occupancy of meis1‐pbx and Hox‐pbx Complexes on Promoters of Leukemia‐associated Genes,” Molecular and Cellular Biology 26 (2006): 3902–3916, 10.1128/MCB.26.10.3902-3916.2006. PMC1488994 16648484

[31] A. Inbal , N. Halachmi , C. Dibner , D. Frank , and A. Salzberg , “Genetic Evidence for the Transcriptional‐activating Function of Homothorax During Adult Fly Development,” Development 128 (2001): 3405–3413, 10.1242/dev.128.18.3405. 11566847

[32] C. Hyman‐Walsh , G. A. Bjerke , and D. Wotton , “An Autoinhibitory Effect of the Homothorax Domain of Meis2,” FEBS Journal 277 (2010): 2584–2597. 10.1111/j.1742-464X.2010.07668.x PMC4306237 20553494

[33] S. K. Choe , P. Lu , M. Nakamura , J. Lee , and C. G. Sagerström , “Meis Cofactors Control HDAC and CBP Accessibility at Hox‐regulated Promoters During Zebrafish Embryogenesis,” Developmental Cell 17 (2009): 561–567, 10.1016/j.devcel.2009.08.007. PMC2768649 19853569

[34] J. J. Louw , A. Corveleyn , Y. Jia , G. Hens , M. Gewillig , and K. Devriendt , “MEIS2 Involvement in Cardiac Development, Cleft Palate, and Intellectual Disability,” American Journal of Medical Genetics Part A 167a (2015): 1142–1146, 10.1002/ajmg.a.36989. 25712757

[35] A. Fujita , B. Isidor , H. Piloquet , et al., “De Novo MEIS2 Mutation Causes Syndromic Developmental Delay With Persistent Gastro‐esophageal Reflux,” Journal of Human Genetics 61 (2016): 835–838, 10.1038/jhg.2016.54. 27225850

[36] A. Gangfuß , G. Yigit , J. Altmüller , et al., “Intellectual Disability Associated With Craniofacial Dysmorphism, Cleft Palate, and Congenital Heart Defect Due to a De Novo MEIS2 Mutation: A Clinical Longitudinal Study,” American Journal of Medical Genetics Part A 185 (2021): 1216–1221, 10.1002/ajmg.a.62070. 33427397

[37] S. B. Ng , A. W. Bigham , K. J. Buckingham , et al., “Exome Sequencing Identifies MLL2 Mutations as a Cause of Kabuki Syndrome,” Nature Genetics 42 (2010): 790–793, 10.1038/ng.646. PMC2930028 20711175

[38] D. Hu , X. Gao , M. A. Morgan , H. M. Herz , E. R. Smith , and A. Shilatifard , “The MLL3/MLL4 Branches of the COMPASS Family Function as Major Histone H3K4 Monomethylases at Enhancers,” Molecular and Cellular Biology 33 (2013): 4745–4754, 10.1128/MCB.01181-13. PMC3838007 24081332

[39] S. Y. Ang , A. Uebersohn , C. I. Spencer , et al., “KMT2D regulates Specific Programs in Heart Development via Histone H3 Lysine 4 Di‐methylation,” Development 143 (2016): 810–821, 10.1242/dev.132688. PMC4813342 26932671

[40] B. J. Aubrey , J. A. Cutler , W. Bourgeois , et al., “IKAROS and MENIN Coordinate Therapeutically Actionable Leukemogenic Gene Expression in MLL‐r Acute Myeloid Leukemia,” Nature Cancer 3 (2022): 595–613, 10.1038/s43018-022-00366-1. PMC9404532 35534777

[41] L. O'Connor , J. Gilmour , and C. Bonifer , “The Role of the Ubiquitously Expressed Transcription Factor Sp1 in Tissue‐specific Transcriptional Regulation and in Disease,” Yale Journal of Biology and Medicine 89 (2016): 513–525. PMC5168829 28018142

[42] D. Dolfini , C. Imbriano , and R. Mantovani , “The Role(s) of NF‐Y in Development and Differentiation,” Cell Death & Differentiation 32 (2025): 195–206, 10.1038/s41418-024-01388-1. PMC11802806 39327506

[43] A. M. Näär , B. D. Lemon , and R. Tjian , “Transcriptional Coactivator Complexes,” Annual Review of Biochemistry 70 (2001): 475–501, 10.1146/annurev.biochem.70.1.475. 11395415

[44] L. F. Soto , Z. Li , C. S. Santoso , et al., “Compendium of Human Transcription Factor Effector Domains,” Molecular Cell 82 (2022): 514–526, 10.1016/j.molcel.2021.11.007. PMC8818021 34863368

[45] A. Erijman , L. Kozlowski , S. Sohrabi‐Jahromi , et al., “A High‐Throughput Screen for Transcription Activation Domains Reveals Their Sequence Features and Permits Prediction by Deep Learning,” Molecular Cell 78 (2020): 890–902.e6, 10.1016/j.molcel.2020.04.020. PMC7275923 32416068

[46] M. V. Staller , A. S. Holehouse , D. Swain‐Lenz , R. K. Das , R. V. Pappu , and B. A. Cohen , “A High‐Throughput Mutational Scan of an Intrinsically Disordered Acidic Transcriptional Activation Domain,” Cell Systems 6 (2018): 444–455.e6, 10.1016/j.cels.2018.01.015. PMC5920710 29525204

[47] C. N. J. Ravarani , T. Y. Erkina , G. De Baets , D. C. Dudman , A. M. Erkine , and M. M. Babu , “High‐Throughput Discovery of Functional Disordered Regions: Investigation of Transactivation Domains,” Molecular Systems Biology 14 (2018): MSB188190, 10.15252/msb.20188190. PMC5949888 29759983

[48] C. D. Arnold , F. Nemčko , A. R. Woodfin , et al., “A High‐Throughput Method to Identify Trans‐Activation Domains Within Transcription Factor Sequences,” The EMBO Journal 37 (2018): 98896, 10.15252/embj.201798896. PMC6092621 30006452

[49] N. Alerasool , H. Leng , Z. Y. Lin , A. C. Gingras , and M. Taipale , “Identification and Functional Characterization of Transcriptional Activators in Human Cells,” Molecular Cell 82 (2022): 677–695.e7, 10.1016/j.molcel.2021.12.008. 35016035

[50] J. Tycko , N. DelRosso , G. T. Hess , et al., “High‐Throughput Discovery and Characterization of Human Transcriptional Effectors,” Cell 183 (2020): 2020–2035.e16, 10.1016/j.cell.2020.11.024. PMC8178797 33326746

[51] Y. Kang , N. R. Patel , C. Shively , et al., “Dual Threshold Optimization and Network Inference Reveal Convergent Evidence From TF Binding Locations and TF Perturbation Responses,” Genome Research 30 (2020): 459–471, 10.1101/gr.259655.119. PMC7111528 32060051

[52] S. Joo , M. H. Wang , G. Lui , et al., “Common Ancestry of Heterodimerizing TALE Homeobox Transcription Factors Across Metazoa and Archaeplastida,” BMC Biology 16 (2018): 136, 10.1186/s12915-018-0605-5. PMC6219170 30396330

[53] K. Mukherjee and T. R. Bürglin , “Comprehensive Analysis of Animal TALE Homeobox Genes: New Conserved Motifs and Cases of Accelerated Evolution,” Journal of Molecular Evolution 65 (2007): 137–153, 10.1007/s00239-006-0023-0. 17665086

[54] K. Kawasaki and T. Fukaya , “Regulatory Landscape of Enhancer‐mediated Transcriptional Activation,” Trends in Cell Biology 34 (2024): 826–837, 10.1016/j.tcb.2024.01.008. 38355349

[55] H. Mohammed , C. Taylor , G. D. Brown , E. K. Papachristou , J. S. Carroll , and C. S. D'Santos , “Rapid Immunoprecipitation Mass Spectrometry of Endogenous Proteins (RIME) for Analysis of Chromatin Complexes,” Nature Protocols 11 (2016): 316–326, 10.1038/nprot.2016.020. 26797456

[56] R. M. Sears , D. G. May , and K. J. Roux , “(2019). BioID as a Tool for Protein‐Proximity Labeling in Living Cells,” Methods in Molecular Biology 2012 (2012): 299–313. 10.1007/978-1-4939-9546-2_15 PMC6583792 31161514

[57] L. Currey , S. Thor , and M. Piper , “TEAD Family Transcription Factors in Development and Disease,” Development 148 (2021): dev196675, 10.1242/dev.196675. 34128986

[58] R. Mevel , J. E. Draper , M. Lie‐a‐Ling , V. Kouskoff , and G. Lacaud , “RUNX Transcription Factors: Orchestrators of Development,” Development 146 (2019): dev148296, 10.1242/dev.148296. 31488508

[59] M. Tremblay , O. Sanchez‐Ferras , and M. Bouchard , “GATA Transcription Factors in Development and Disease,” Development 145 (2018): dev164384, 10.1242/dev.164384. 30348673

[60] L. Mahendrawada , L. Warfield , R. Donczew , and S. Hahn , “Low Overlap of Transcription Factor DNA Binding and Regulatory Targets,” Nature 642 (2025): 796–804, 10.1038/s41586-025-08916-0. PMC12882699 40240607

[61] N. DelRosso , J. Tycko , P. Suzuki , et al., “Large‐scale Mapping and Mutagenesis of Human Transcriptional Effector Domains,” Nature 616 (2023): 365–372, 10.1038/s41586-023-05906-y. PMC10484233 37020022

[62] R. Martinez‐Corral , D. Friedrich , R. Frömel , L. Velten , J. Gunawardena , and A. H. DePace , “Emergence of Activation or Repression in Transcriptional Control Under a Fixed Molecular Context,” Proc Natl Acad Sci USA 122 (2025): e2413715122, 10.1073/pnas.2413715122. PMC12501156 40982681

[63] M. Losa , I. Barozzi , M. Osterwalder , et al., “A Spatio‐temporally Constrained Gene Regulatory Network Directed by PBX1/2 Acquires Limb Patterning Specificity via HAND2,” Nature Communications 14 (2023): 3993, 10.1038/s41467-023-39443-z. PMC10325989 37414772

[64] C. A. Berkes , D. A. Bergstrom , B. H. Penn , K. J. Seaver , P. S. Knoepfler , and S. J. Tapscott , “Pbx Marks Genes for Activation by MyoD Indicating a Role for a Homeodomain Protein in Establishing Myogenic Potential,” Molecular Cell 14 (2004): 465–477, 10.1016/S1097-2765(04)00260-6. 15149596

[65] W. Stanney , F. Ladam , I. J. Donaldson , et al., “Combinatorial Action of NF–Y and TALE at Embryonic Enhancers Defines Distinct Gene Expression Programs During Zygotic Genome Activation in Zebrafish,” Developmental Biology 459 (2020): 161–180, 10.1016/j.ydbio.2019.12.003. PMC7080602 31862379

[66] F. Ladam , W. Stanney , I. J. Donaldson , O. Yildiz , N. Bobola , and C. G. Sagerström , “TALE Factors Use Two Distinct Functional Modes to Control an Essential Zebrafish Gene Expression Program,” Elife 7 (2018): 36144, 10.7554/eLife.36144. PMC6023610 29911973

[67] L. Magnani , D. K. Patten , V. T. M. Nguyen , et al., “The Pioneer Factor PBX1 Is a Novel Driver of Metastatic Progression in ERα‐positive Breast Cancer,” Oncotarget 6 (2015): 21878–21891, 10.18632/oncotarget.4243. PMC4673133 26215677

[68] H. Wang , Y. Yang , J. Liu , and L. Qian , “Direct Cell Reprogramming: Approaches, Mechanisms, and Progress,” Nature Reviews Molecular Cell Biology 22 (2021): 410–424, 10.1038/s41580-021-00335-z. PMC8161510 33619373

